# Contemporary use of guideline‐based higher potency P2Y12 receptor inhibitor therapy in patients with moderate‐to‐high risk non‐ST‐segment elevation myocardial infarction: Results from the Canadian ACS reflective II cross‐sectional study

**DOI:** 10.1002/clc.23618

**Published:** 2021-05-13

**Authors:** Ashish Patel, Shaun G. Goodman, Mary Tan, Neville Suskin, Robert McKelvie, Andrew L. Mathew, Sohrab Lutchmedial, Payam Dehghani, Andrea J. Lavoie, Thao Huynh, Shahar Lavi, Roger Philipp, Razi Khan, Andrew T. Yan, Sam Radhakrishnan, Tara Sedlak, Nathan Brunner, Hahn Hoe Kim, Tomas Cieza, Saleem Kassam, Christopher B. Fordyce, Michael Heffernan, Sean Jedrzkiewicz, Mina Madan, Shaheeda Ahmed, Colin Barry, Jean‐Pierre Dery, Akshay Bagai, for the Canadian ACS Reflective II Investigators

**Affiliations:** ^1^ Terrence Donnelly Heart Centre, St Michael's Hospital University of Toronto Toronto Canada; ^2^ Canadian Heart Research Centre Toronto Canada; ^3^ St Joseph's Health Care London Western University, Lawson Research Institute London Canada; ^4^ University Hospital, London Health Sciences Centre London Canada; ^5^ New Brunswick Heart Centre Saint John Canada; ^6^ Regina General Hospital ‐ Prairie Vascular Research Network Regina Canada; ^7^ McGill University Health Centre Montreal Canada; ^8^ Royal Columbian Hospital, Keary Medical Centre New Westminster Canada; ^9^ Sunnybrook Health Sciences Centre University of Toronto Toronto Canada; ^10^ Division of Cardiology University of British Columbia Vancouver Canada; ^11^ St Mary's Regional Cardiac Centre Kitchener Canada; ^12^ Institut universitaire de cardiologie et de pneumologie de Québec – Université Laval Quebec City Canada; ^13^ Rouge Valley Health System Toronto Canada; ^14^ Halton Healthcare Oakville Hospital Oakville Canada

**Keywords:** acute coronary syndrome, pharmacology

## Abstract

**Background:**

After myocardial infarction, guidelines recommend higher‐potency P2Y12 receptor inhibitors, namely ticagrelor and prasugrel, over clopidogrel.

**Hypothesis:**

We aimed to determine the contemporary use of higher‐potency antiplatelet therapy in Canadian patients with non‐ST‐elevation myocardial infarction (NSTEMI).

**Methods:**

A total of 684 moderate‐to‐high risk NSTEMI patients were enrolled in the prospective Canadian ACS Reflective II registry at 12 Canadian hospitals and three clinics in five provinces between July 2016 and May 2018. Multivariable logistic regression modeling was performed to assess factors independently associated with higher‐potency P2Y12 receptor inhibitor use at discharge.

**Results:**

At hospital discharge, 78.3% of patients were treated with a P2Y12 receptor inhibitor. Among patients discharged on a P2Y12 receptor inhibitor, use of higher‐potency P2Y12 receptor inhibitor was 61.4%. After adjustment, treatment in‐hospital with PCI (OR 4.48, 95%CI 3.34–6.03, *p* < .0001) was most strongly associated with higher use of higher‐potency P2Y12 receptor inhibitor, while oral anticoagulant use at discharge (OR 0.03, 95%CI 0.01–0.12, *p* < .0001), and atrial fibrillation (OR 0.40, 95%CI 0.17–0.98, *p* = .046) were most strongly associated with lower use of higher‐potency P2Y12 receptor inhibitor. Use of higher‐potency P2Y12 receptor inhibitor varied across provinces (range, 21.6%–78.9%).

**Discussion:**

In contemporary Canadian practice, approximately 60% of moderate‐to‐high risk NSTEMI patients discharged on a P2Y12 receptor inhibitor are treated with a higher‐potency P2Y12 receptor inhibitor. In addition to factors that increase risk of bleeding, interprovincial differences in practice patterns were associated with use of higher‐potency P2Y12 receptor inhibitor at discharge. Opportunities remain for further optimization of evidence‐based, guideline‐recommended antiplatelet therapy use.

## 1 BACKGROUND

Dual antiplatelet therapy (DAPT) with aspirin and an oral P2Y12 receptor inhibitor is the cornerstone of treatment to prevent recurrent cardiovascular events after myocardial infarction (MI). The efficacy and safety of higher potency P2Y12 receptor inhibitors ticagrelor and prasugrel compared with clopidogrel were demonstrated in rigorous clinical trials, now approximately one decade ago.^1^, ^2^ As such the Canadian Cardiovascular Society/Canadian Association of Interventional Cardiology guidelines for the Use of Antiplatelet Therapy recommend the use of higher potency P2Y12 receptor inhibitors ticagrelor and prasugrel preferentially over clopidogrel.^3^ While two Canadian observational studies of MI patients demonstrated temporal increases in P2Y12 receptor inhibitor use acutely and at discharge compared with prior national experience,^4^, ^5^ guideline‐recommended use of the higher potency P2Y12 receptor inhibitors remained low, particularly among patients not undergoing PCI and in those with a diagnosis of NSTEMI. The Canadian Acute Coronary Syndrome (ACS) Reflective Program was designed to assess the appropriate use of oral antiplatelet therapies, with a primary aim to evaluate care gaps that exist between evidence‐based guideline recommendations and real world practice. Here we report the contemporary in hospital and discharge use of guideline recommended higher potency P2Y12 receptor inhibitors in a moderate‐to‐high risk Canadian NSTEMI population, and identify opportunities where higher potency DAPT may be of potential benefit in the management of such patients.

## 2 METHODS

The Canadian ACS Reflective II, a prospective Quality Enhancement Research Initiative (QuERI) is a knowledge translation program designed to evaluate physician decision‐making and choice of guideline‐recommended antiplatelet therapies in the management of NSTEMI patients. The program was conducted by the Canadian Heart Research Centre (CHRC), a not‐for‐profit academic research organization, with the oversight of a steering committee comprised of four Canadian cardiologists. The program objectives were: (1) to identify and describe NSTEMI patients who do not receive guideline‐recommended DAPT (ASA and P2Y12 receptor inhibitor), specifically higher potency P2Y12 receptor inhibitor, and (2) to identify opportunities where DAPT may be of potential benefit in the management of moderate‐to‐high risk patients. The local research ethics board at each participating center approved the study. Informed consent was obtained from all participating subjects.

Both academic and community based cardiologists and internists in Canada were invited to participate in the project. These included physicians who participated in the original ACS Reflective program as well as additional physicians who have participated previously in other ACS studies. Each physician was asked to enroll approximately 15–20 subjects each. Patient inclusion criteria were: age ≥ 18 years of age, hospitalization with NSTEMI (positive biomarker[s] for myocardial necrosis), and moderate‐to‐high risk NSTEMI (defined as ≥1 of the following criteria: ischemic ST‐segment changes; age ≥ 60 years; previous MI or coronary artery bypass grafting (CABG); coronary artery disease with stenosis ≥50% in ≥2 vessels; previous ischemic stroke, transient ischemic attack, carotid stenosis of ≥50% or cerebral revascularization; diabetes; peripheral arterial disease; or chronic renal dysfunction with creatinine clearance <60 ml/min/1.73m^2^). Exclusion criteria included STEMI, unstable angina (i.e., ACS without positive cardiac biomarker[s] for myocardial necrosis), and ongoing participation in a research study where the oral antithrombotic therapy (antiplatelet and/or anticoagulant) were unknown or not approved for clinical use.

Participating physicians provided data on post‐ACS patient management through the use of a case report form (CRF). The CRF included questions on demographics, key presenting characteristics, information regarding diagnosis, study eligibility verification, prior medical history, in‐hospital management (including medical and revascularization therapies), and treatment received at discharge or hospital transfer. This included highlighting instances where management was not consistent with the guidelines or evidence‐based recommendations (care gaps) and identification of the reason(s) why such differences occurred. These were presented to the physician as standardized options with availability for free text entry. In hospital and at discharge treatment decisions were all made by the treating physician and not mandated by the study. Individual physician data were aggregated. The primary criterion for evaluation was the proportion of patients on higher potency DAPT as recommended by the CCS antiplatelet guidelines at hospital discharge.

The sample size of the enrolled population was determined on the basis of feasibility and cost. Descriptive statistics were summarized as medians with 25th and 75th percentiles for continuous variables and as percentages for categorical variables. Differences between groups were compared by use of Kruskal‐Wallis test for continuous variables and Pearson's chi‐squared test for categorical variables.

Multivariable logistic regression models were developed to assess factors independently associated with (1) P2Y12 receptor inhibitor at discharge and (2) High potency P2Y12 receptor inhibitor at discharge (among those discharged on P2Y12 receptor inhibitor). The following variables were considered: age, sex, weight, province, diabetes, hypertension, smoking, prior MI, prior PCI, prior CABG, atrial fibrillation, prior stroke, CKD (defined as eGFR<60), peripheral arterial disease, ECG on presentation (ST elevation, ST depression, T wave, non‐specific), angiogram in hospital, PCI in hospital, CABG in hospital, OAC at discharge, stent thrombosis in hospital, and blood transfusion in hospital. To account for the clustering of patients within different hospitals, we performed a generalized estimating equations (GEE) model. The working correlation structure selected was based on its lowest quasi‐likelihood under the independence model criterion (QIC). Intraclass correlation coefficient (ICC) was determined to estimate the degree in variation in the P2Y12 receptor inhibitor use at discharge, as well as high potency P2Y12 receptor inhibitor use at discharge, accounted for by clustering within hospitals. Adjusted odds ratio (OR) with 95% confidence intervals (CI) are presented. A value of *p* < .05 was considered significant for all tests. All statistical analyses were performed in SAS software version 9.4 (SAS Institute, Cary, NC).

## 3 RESULTS

A total of 684 patients were enrolled by 52 cardiologists in 12 hospitals and 3 clinics across 5 Canadian provinces from July 2016 to May 2018 (see supplementary appendix for complete list of physicians); three patients died during index hospitalization and were excluded from the analysis. Off the 681 patients in the analysis cohort, 105 (15.4%) were from British Columbia, 93 (13.7%) were from New Brunswick, 357 (52.4%) were from Ontario, 58 (8.5%) were from Quebec and 68 (10%) were from Saskatchewan.

The median (25th, 75th percentile) age of the study population was 67 (59, 75) years, 69.7% were male, 33.3% had diabetes, 22.8% had prior MI, 18.0% had prior PCI, 8.9% had prior CABG, 5.3% had prior heart failure, 5.6% had peripheral arterial disease, 4.8% had prior stroke, and 7.3% had atrial fibrillation. On presentation, 12.2% had Killip >1 heart failure and 1.3% had cardiac arrest. Transient ST‐segment elevation was present in 8.8%, and ST segment depression was present in 27.5%. During index hospitalization, 87.3% underwent coronary angiography, while 61.0% and 19.6% underwent PCI and CABG, respectively; 20.2% of the study population was managed medically and did not undergo revascularization during index hospitalization. Among patients undergoing PCI, 97.1% received drug‐eluting stents.

In the first 24 h of hospitalization, 98.5% of patients received aspirin and 91.8% were treated with at least one P2Y12 receptor inhibitor (60.9% ticagrelor, 0.8% prasugrel, 42.8% clopidogrel). Two patients (0.3%) had stent thrombosis, 9 (1.3%) had major bleeding and 32 (4.7%) received blood transfusion.

At hospital discharge, 97.1% of patients were treated with aspirin and 78.3% were treated with a P2Y12 receptor inhibitor (ticagrelor 60.6%, prasugrel 0.8%, clopidogrel 38.6%). Oral anticoagulation use at discharge was 10.6% (vitamin K antagonist 30.6%, apixaban 30.6%, rivaroxaban 33.3%, and dabigatran 5.6%). Compared with patients discharged on a P2Y12 receptor inhibitor, patients not discharged on a P2Y12 receptor inhibitor had a higher prevalence of atrial fibrillation (14.2% vs. 5.4%, *p* < .001), and were more likely to be treated with an OAC (25.7% vs. 6.4%, *p* < .0001) (Table 1). Use of higher potency P2Y12 receptor inhibitor among patients without contraindication for higher potency P2Y12 receptor inhibitor or high bleeding risk such as OAC use was 71.1%. Use of P2Y12 receptor inhibitor at discharge was 98.1% among patients treated in‐hospital with PCI, 25.6% among patients treated in‐hospital with CABG, and 67.2% among patients treated medically. After adjustment, treatment in hospital with PCI was most strongly associated with discharge on a P2Y12 receptor, while treatment in hospital with CABG and use of OAC at discharge were most strongly associated with not being discharged on a P2Y12 receptor inhibitor (Table 2). Approximately 3% of the variation in the use of P2Y12 receptor inhibitor use at discharge was accounted for by differences in P2Y12 receptor inhibitor use at discharge between hospitals (*p* = .25).

**TABLE 1:** Characteristics of study patients stratified by discharge on a P2Y12 receptor inhibitor versus discharge on No P2Y12 receptor inhibitor

	Any P2Y12 at discharge (*n* = 533, 78.3%)	No P2Y12 at discharge (*n* = 148, 21.7%)	*p* value
Demographics			
Age, years^a^	67 (59, 75)	68 (61, 75)	.51
Age ≥ 75 years	25.9%	27.7%	.66
Sex, male	69.2%	73.0%	.38
Weight, kg^a^	82 (70, 94)	82 (71, 93)	.89
Medical history			
Diabetes	32.8%	35.1%	.60
Hypertension	67.5%	68.2%	.86
Dyslipidemia	58.8%	63.5%	.30
Smoking, current or past	57.7%	58.9%	.80
Prior myocardial infarction	23.1%	21.6%	.71
Prior percutaneous coronary intervention	18.4%	15.5%	.43
Prior coronary artery bypass grafting	9.4%	6.8%	.32
Prior heart failure	4.9%	6.8%	.37
Peripheral arterial disease	5.1%	7.4%	.27
Atrial fibrillation	5.4%	14.2%	.0003
Prior stroke	5.1%	4.1%	.61
Presentation characteristics			
Heart rate, bpm^a^	75 (66, 87)	81 (68, 90)	.08
Systolic blood pressure, mmHg^a^	139 (122, 160)	141 (127, 157)	.47
Killip >1 CHF	10.0%	19.9%	.002
Cardiac arrest	1.3%	1.4%	1.00
ECG on presentation			
Transient ST‐segment elevation	9.6%	6.1%	.19
ST‐segment depression	25.1%	35.1%	.02
T wave inversion	23.3%	23.6%	.92
Non‐specific ST and T wave abnormality	24.0%	23.6%	.93
Normal (no ST segment or T wave abnormality)	27.8%	23.6%	.32
Invasive procedures			
Coronary angiography	85.7%	92.6%	.03
Percutaneous coronary intervention	76.5%	5.4%	<.0001
Coronary artery bypass grafting	6.4%	66.9%	<.0001
Anticoagulant use			
Oral anticoagulant at discharge	6.4%	25.7%	<.0001

^a^ Median (25th, 75th) percentiles.

**TABLE 2:** Factors associated with discharge on a P2Y12 receptor inhibitor

Parameter	OR (95%CI)	*p* value
Age (per 1 year)	1.01 (0.99–1.04)	.41
Sex, female	0.83 (0.46–1.47)	.52
Weight (per 1 kg)	1.0 (0.98–1.02)	.75
Province ‐ Ontario (reference)		
British Columbia	0.79 (0.33–1.87)	.59
New Brunswick	1.18 (0.70–2.0)	.54
Quebec	0.65 (0.12–3.56)	.62
Saskatchewan	0.46 (0.23–0.90)	.02
Diabetes	1.53 (0.75–3.13)	.25
Hypertension	1.78 (1.03–3.10)	.04
Smoking	1.24 (0.68–2.23)	.48
Prior myocardial infarction	1.01 (0.51–1.98)	.98
Prior percutaneous coronary intervention	1.01 (0.56–1.81)	.97
Prior coronary artery bypass grafting	1.20 (0.40–3.60)	.74
Atrial fibrillation	0.56 (0.21–1.52)	.26
Prior stroke	1.86 (0.76–4.57)	.18
Chronic kidney disease	0.59 (0.31–1.13)	.11
Peripheral arterial disease	0.85 (0.21–3.34)	.81
ECG findings		
ST‐elevation	1.38 (0.80–2.39)	.25
ST‐depression	0.79 (0.49–1.28)	.34
T wave changes	1.23 (0.60–2.51)	.57
Non‐specific changes	1.32 (0.50–3.47)	.57
Cardiac catheterization in hospital	0.41 (0.11–1.43)	.16
Percutaneous coronary intervention in hospital	27.9 (14.7–53.1)	<.0001
Coronary artery bypass grafting in hospital	0.10 (0.03–0.31)	<.0001
Oral anticoagulant use at discharge	0.15 (0.08–0.28)	<.0001
Blood transfusion in hospital	1.94 (0.61–6.20)	.26

Compared with patients receiving clopidogrel at hospital discharge, patients receiving higher potency P2Y12 receptor inhibitor at hospital discharge were younger, less likely to be female, have atrial fibrillation, prior PCI, prior stroke, and prior heart failure (Table 3). Among patients treated with P2Y12 receptor inhibitor at hospital discharge, use of higher potency P2Y12 receptor inhibitor was 67.7% among patients treated in‐hospital with PCI, was 47.1% among patients treated in‐hospital with CABG, and 39.1% among patients treated medically. OAC use was 15.5% among patients treated with clopidogrel at hospital discharge vs. 0.6% among patients treated with ticagrelor/prasugrel (*p* < .0001). There were also interprovincial differences in rates of higher potency P2Y12 receptor inhibitor use (British Columbia 78.9%, New Brunswick 21.6%, Ontario 69.4%, Quebec 49.0%, Saskatchewan 61.2%).

**TABLE 3:** Characteristics of study patients discharged on higher potency P2Y12 receptor inhibitor versus discharge on Clopidogrel

	Clopidogrel at discharge (*n* = 206, 38.6%)	Ticagrelor/Prasugrel at discharge (*n* = 327, 61.4%)	*p* value
Demographics			
Age, years^a^	70 (62, 79)	65 (57, 73)	<.0001
Age ≥ 75 years	37.4%	18.7%	<.0001
Sex, male	64.1%	72.5%	.04
Weight, kg^a^	80 (67, 93)	83 (72, 94)	.11
Medical history			
Diabetes	37.9%	29.7%	.050
Hypertension	75.7%	62.3%	.001
Dyslipidemia	63.6%	55.8%	.08
Smoking, current or past	54.6%	59.6%	.26
Prior myocardial infarction	26.7%	20.8%	.12
Prior percutaneous coronary intervention	23.3%	15.3%	.02
Prior coronary artery bypass grafting	13.1%	7.0%	.02
Prior heart failure	9.7%	1.8%	<.0001
Peripheral arterial disease	5.3%	4.9%	.82
Atrial fibrillation	11.7%	1.5%	<.0001
Prior stroke	7.8%	3.4%	.02
Presentation characteristics			
Heart rate, bpm^a^	77 (66, 92)	75 (66, 86)	.17
Systolic blood pressure, mmHg^a^	139 (119, 160)	138 (124, 160)	.43
Killip >1 CHF	13.2%	8.0%	.05
Cardiac arrest	0.5%	1.8%	.26
ECG on presentation			
Transient ST‐segment elevation	9.7%	9.5%	.93
ST‐segment depression	24.8%	25.4%	.87
T wave inversion	18.9%	26.0%	.06
Non‐specific ST and T wave abnormality	25.2%	23.2%	.60
Normal (no ST segment or T wave abnormality)	26.7%	28.4%	.66
Invasive procedures			
Coronary angiography	85.4%	85.9%	.87
Percutaneous coronary intervention	64.1%	84.4%	<.0001
Coronary artery bypass grafting	8.7%	4.9%	.08
Anticoagulant use			
Oral anticoagulant at discharge	15.5%	0.6%	<.0001

^a^ Median (25th, 75th) percentiles.

After adjustment, among patients discharged on a P2Y12 receptor inhibitor, treatment in‐hospital with PCI was most strongly associated with higher use of higher potency P2Y12 receptor inhibitor, while OAC use at discharge, atrial fibrillation, chronic kidney disease, and prior PCI were associated with lower use of higher potency P2Y12 receptor inhibitor (Table 4). There were also interprovincial differences in use of higher potency P2Y12 receptor inhibitor at discharge. With Ontario serving as reference, British Columbia was associated with higher use of higher potency P2Y12 receptor inhibitors, while New Brunswick and Quebec were associated with lower use of higher potency P2Y12 receptor inhibitors. Approximately 20% of the variation in the use of high potency P2Y12 receptor inhibitor use was accounted for by differences in high potency P2Y12 receptor inhibitor use between hospitals (*p* = .02).

**TABLE 4:** Factors associated with discharge on a higher potency P2Y12 receptor inhibitor versus Clopidogrel among patients discharged on a P2Y12 receptor inhibitor

Parameter	OR (95%CI)	*p* value
Age (per 1 year)	0.98 (0.95–1.00)	.09
Sex, female	0.72 (0.39–1.32)	.29
Weight (per 1 kg)	1.00 (0.98–1.02)	.86
Province – Ontario (reference)		
British Columbia	1.63 (1.34–1.98)	<.0001
New Brunswick	0.08 (0.07–0.10)	<.0001
Quebec	0.29 (0.09–0.98)	.047
Saskatchewan	0.63 (0.30–1.30)	.21
Diabetes	0.83 (0.56–1.23)	.36
Hypertension	0.97 (0.57–1.65)	.90
Smoking	1.03 (0.57–1.87)	.91
Prior myocardial infarction	1.58 (0.92–2.72)	.10
Prior percutaneous coronary intervention	0.46 (0.23–0.92)	.03
Prior coronary artery bypass grafting	0.97 (0.47–2.00)	.93
Atrial fibrillation	0.40 (0.17–0.98)	.046
Prior stroke	0.80 (0.25–2.56)	.71
Chronic kidney disease	0.42 (0.18–0.95)	.04
Peripheral arterial disease	1.84 (0.89–3.84)	.10
ECG findings		
ST‐elevation	0.91 (0.32–2.61)	.87
ST‐depression	1.08 (0.61–1.90)	.79
T wave	1.38 (0.87–2.19)	.17
Non‐specific	0.94 (0.41–2.17)	.88
Cardiac catheterization in hospital	0.44 (0.19–1.00)	.049
Percutaneous coronary intervention in hospital	4.48 (3.34–6.03)	<.0001
Coronary artery bypass grafting in hospital	0.98 (0.24–4.11)	.98
Oral anticoagulation use at discharge	0.03 (0.01–0.12)	<.0001
Stent thrombosis in hospital	0.46 (0.06–3.28)	.44
Blood transfusion in hospital	0.44 (0.09–2.06)	.29

Reasons (not mutually exclusive) identified by physicians for treatment with clopidogrel and not a higher potency P2Y12 receptor inhibitor at the time of discharge was provided for 194 of the 206 patients discharged on clopidogrel (94.2%). These include perceived high risk for bleeding (29.4%), physician preference (36.6%), in‐hospital CABG (4.1%), concomitant OAC use (9.3%), older age (13.9%), renal dysfunction (6.2%) and affordability issues (5.7%).

## 4 DISCUSSION

In this assessment of contemporary Canadian practice, approximately 4 out of 5 moderate‐to‐high risk NSTEMI patients were treated with a P2Y12 receptor inhibitor at hospital discharge. Rates of discharge P2Y12 receptor inhibitor varied markedly by revascularization type, with near universal use at discharge among PCI treated patients, but relatively low use among CABG and medically treated patients. Among those treated with P2Y12 receptor inhibitor at discharge, approximately 60% received a higher potency P2Y12 receptor inhibitor while 40% received clopidogrel. In addition to factors associated with higher risk of bleeding (e.g. treatment with OAC), physician practice patterns with preference for one drug versus another was self‐identified for non‐use of a higher potency P2Y12 receptor inhibitor.

Compared with prior Canadian observational cohorts from 2011 to 2013,^4^ we noted an increase in early but not discharge use of P2Y12 receptor inhibitor. This increase in early P2Y12 receptor inhibitor was solely due to an increase in early ticagrelor use, while use of prasugrel was unchanged and remained infrequent. Prasugrel, while associated with improved cardiovascular outcomes compared with clopidogrel in the TRITON‐TIMI 38 study, was not initiated until PCI.^2^ The ACCOAST trial seeking to determine the optimal timing of prasugrel in patients with NSTEMI scheduled for coronary angiography was stopped prematurely, due to an increase in bleeding in patients with a 30 mg loading dose before angiography.^6^ In contrast, the PLATO trial enrolled patients with the whole spectrum of ACS, regardless of initial invasive strategy. In addition, approximately half the patients were pretreated with clopidogrel prior to randomization. The benefit of ticagrelor over clopidogrel in reducing ischemic events and total mortality in the NSTEMI cohort of PLATO patients was consistent with the overall PLATO trial results.^7^

Rates of P2Y12 receptor inhibitor use at discharge were similar compared with historic Canadian cohorts.^4^ However, only one quarter of current patients not discharged on a P2Y12 receptor inhibitor were treated with OAC upon discharge, with the remaining non treatment with P2Y12 receptor inhibitor explained predominantly by non‐use among CABG and medically treated patients. Rates of P2Y12 receptor inhibitor at discharge were low (67.2%) among medically managed patients and particularly low (25.6%) among CABG treated patients. The benefit of addition of clopidogrel to aspirin among NSTEMI patients in the CURE trial was observed irrespective of whether patients underwent revascularization.^8^ Similarly, the benefit of ticagrelor over clopidogrel among NSTEMI patients in the PLATO trial was independent of type of revascularization during the initial 10 days.^7^ In the subgroup of PLATO patients undergoing CABG within 7 days after the last study drug intake, ticagrelor compared with clopidogrel was associated with a substantial reduction in total and CV mortality without excess risk of CABG‐related bleeding.^9^ Accordingly, the noted benefit of addition of clopidogrel or ticagrelor to aspirin over aspirin alone in CABG treated or medical therapy alone treated ACS patients was endorsed in the 2012 CCS Antiplatelet guidelines which recommend ticagrelor for moderate‐to‐high risk NSTEMI patients managed with either PCI, CABG or medical therapy alone^10^; clopidogrel is recommended for patients who are not eligible for ticagrelor. Prasugrel on the other hand, was not associated with reduction in ischemic outcomes compared with clopidogrel among patients with unstable angina or NSTEMI who did not undergo revascularization.^11^ Since February 2020, prasugrel is no longer marketed or available in Canada. The 2020 European Society of Cardiology Guidelines for the management of acute coronary syndromes in patients presenting without persistent ST segment elevation also recomment ticagrelor or prasugrel as standard treatment for NSTEMI, with clopidogrel only to be used when prasugrel or ticagrelor are contraindicated, not available or cannot be tolerated due to an unacceptable high bleeding risk.^12^

Although the use of higher potency P2Y12 receptor inhibitors, namely ticagrelor, has increased over time both in early use and at hospital discharge, approximately 40% of moderate‐to‐high risk NSTEMI patients are still discharged on clopidogrel. Use of clopidogrel in patients with contraindication to ticagrelor (e.g. history of intracranial hemorrhage, moderate‐to‐severe hepatic impairment, concomitant use of strong CYP3A4 inhibitors) is reasonable. In addition, higher potency P2Y12 receptor inhibitors are not recommended for use in triple therapy for patients also requiring concomitant OAC given increased risk of bleeding. Use of higher potency P2Y12 receptor in dual pathway with OAC was less than 10% in clinical trials of atrial fibrillation patients with ACS or treatment with PCIPCI.^13^, ^14^, ^15^, ^16^ Thus, the 2018 Canadian Cardiovascular Society/Canadian Association of Interventional Cardiology Focused Update of the Guidelines for the Use of Antiplatelet Therapy recommends use of clopidogrel and not higher potency P2Y12 receptor inhibitors in dual pathway with OAC.^3^ In the current study, bleeding risk avoidance was the predominant stated reason for not using higher potency P2Y12 receptor at hospital discharge. However, even after excluding patients with contraindications for higher potency P2Y12 receptor inhibitors and/or high bleeding risk, use of higher potency P2Y12 receptor inhibitor use was still only 71.1%. Affordability does not appear to be a large reported barrier to higher potency P2Y12 receptor inhibitor use. We found physician practice patterns with preference for one drug versus another to be a self‐identified reason for non‐use of a higher potency P2Y12 receptor inhibitor. This is likely reflected in differences in rates of clopidogrel versus higher potency P2Y12 receptor inhibitor use by patient geography. Use of higher potency P2Y12 receptor inhibitors was significantly lower in New Brunswick and Quebec compared with other provinces despite listing criteria for ticagrelor in these provinces including provision for high risk NSTEMI patients irrespective of revascularization with PCI. Cost of ticagrelor was also relatively similar among provinces. We found that approximately 20% of the variation in use of high potency P2Y12 receptor use at discharge was accounted for by clustering among hospitals. Thus, variability in use of high potency P2Y12 receptor inhibitors among provinces may be more due to differences in in‐hospital pathways and physician preferences than provincial availability and cost. As such, opportunities still remain both geographically and in certain patient subtypes (medically managed NSTEMI) for further optimization of evidence‐based, guideline recommended higher potency P2Y12 receptor inhibitor use in moderate‐to‐high‐risk NSTEMI patients in Canada. Some of these gaps maybe addressed by increasing physician awareness regarding data in support for higher potency P2Y12 receptor inhibitor use among medically managed ACS patients, integration of higher potency P2Y12 receptor inhibitors into hospital treatment algorithm pathways, and resolving discrepancy in practice/hospital formulary between initial admitting hospital and repatriation hospital.

## 5 LIMITATIONS

Several limitations should be considered. Study sample size was small. Physician participation in this study was voluntary, and treatment patterns (including the use of higher potency P2Y12 receptor inhibitors) may therefore not be generalizable to the entire group of Canadian physicians who care for and are involved in antiplatelet decision making for ACS patients. The practice of participating physicians and hospitals is potentially different from that of other physicians and hospitals not participating in the study. In addition, patients enrolled in this study were non‐consecutive possibly also biasing toward enrolling patients more likely to be treated with higher potency P2Y12 receptor inhibitors. Thus, the real‐world rates of both overall and higher potency P2Y12 receptor inhibitor use at discharge, particularly among CABG and medically treated Canadian patients, may be lower than observed in the study cohort. Although we did elucidate reasons for non‐treatment with higher potency P2Y12 receptor inhibitors, information that is usually not available in observational cohort studies, there may be factors beyond those captured on the data collection form which represent unmeasured confounders that contributed to selection of therapies.

## 6 CONCLUSION

Approximately 60% of moderate‐to‐high risk NSTEMI patients discharged on a P2Y12 receptor inhibitor in contemporary Canadian practice are treated with a higher potency P2Y12 receptor inhibitor. In addition to factors associated with higher risk of bleeding, selection for clopidogrel at discharge was associated with not receiving PCI and interprovincial differences in practice patterns. Excluding patients with contraindications for higher potency P2Y12 receptor inhibitors and/or high bleeding risk, use of higher potency P2Y12 receptor inhibitor was still only 71.1%. Opportunities remain geographically, and among medically managed high‐risk NSTEMI patients for further optimization of evidence‐based, guideline‐recommended antiplatelet therapy use.

## CONFLICT OF INTEREST

Ashish Patel: no relevant disclosures. Shaun G. Goodman receives research grant support (e.g., steering committee or data monitoring committee) and/or speaker/consulting honoraria (e.g., advisory boards) from: Amgen, AstraZeneca, Bayer, Boehringer Ingelheim, Bristol Myers Squibb, CSL Behring, Daiichi‐Sankyo, Eli Lilly, Esperion, Fenix Group International, Ferring Pharmaceuticals, GlaxoSmithKline, HLS Therapeutics, Janssen/Johnson & Johnson, Luitpold Pharmaceuticals, Matrizyme, Merck, Novartis, Novo Nordisk A/C, Pfizer, Regeneron, Sanofi, Servier, Tenax Therapeutics; and salary support from the Heart and Stroke Foundation of Ontario/University of Toronto (Polo) Chair, Canadian Heart Research Centre and MD Primer, Canadian VIGOUR Centre, Duke Clinical Research Institute, New York University Clinical Coordinating Centre, and PERFUSE. Mary Tan, no relevant disclosures. Neville Suskin has received speaking/consulting honoraria from Bayer and BMS/Pfizer. Robert McKelvie, no relevant disclosures. Andrew Mathew, no relevant disclosures. Sohrab Lutchmedial has received speaking honoraria from Astra Zeneca, Pfizer, Bayer, Medtronic, Abbott. Payam Dehghani has received speaking/consulting honoraria from AstraZeneca, Bayer, BMS/Pfizer, Boehringer Ingelheim. Andrea Lavoie: no relevant disclosures. Thao Huynh has received research grants and honoraria from Bayer, Sanofi and Astra‐Zeneca. Shahar Lavi, no relevant disclosures. Roger Philipp: no relevant disclosures. Razi Khan: no relevant disclosures. Andrew Yan has received research grant support from Astra Zeneca. Sam Radhakrishnan: no relevant disclosures. Tara Sedlak: no relevant disclosures. Nathan Brunner: no relevant disclosures. Hahn Hoe Kim has received honoraria from Pfizer/BMS, Astra Zeneca, Servier, Bayer. Tomas Cieza has received research grants and/or speaking/consulting honoraria Astra‐Zeneca, Sanofi, Lilly. Saleem Kassam: no relevant disclosures. Christopher Fordyce has received speaking/consulting honoraria from Bayer, Pfizer, Sanofi, Boehringer Ingelheim, Novo Nordisk and research grants from Bayer. Michael Heffernan has received grants/research support from Bayer, Esai, Boehringer Ingelheim, Astra Zeneca, Novartis, and speaking/consulting honoraria from Amgen, Bayer, Boehringer Ingelheim, Astra Zeneca, Pfizer, BMS, Servier, Sanofi, Novartis. Sean Jedrzkiewicz, no relevant disclosures. Mina Madan has received honoraria from Bayer Inc, Pfizer Canada, and Bristol Myers Squibb. Shaheeda Ahmed, no relevant disclosures. Colin Barry, no relevant disclosures. Jean‐Pierre Dery has received speaking/consulting honoraria from Bayer, BMS/Pfizer, Astra, Lilly, Servier, Sanofi, Boehringer, Amgen, Janssen, Novo, Novartis. Akshay Bagai has received speaking/consulting honoraria from AstraZeneca, Bayer, BMS/Pfizer, Servier, HLS Therapeutics and Boehringer Ingelheim.

## Supporting information


**Appendix S1.** Supporting Information.

## Data Availability

The data underlying this article will be shared on reasonable request to the corresponding author.
