# Supplementary material for: Contemporary use of guideline‐based higher potency P2Y12 receptor inhibitor therapy in patients with moderate‐to‐high risk non‐ST‐segment elevation myocardial infarction: Results from the Canadian ACS reflective II cross‐sectional study
Source: Clin Cardiol. 2021 May 13;44(6):839–47. doi: 10.1002/clc.23618 (PMC8207978; doi:10.1002/clc.23618)
Supplement: Supplementary file 1 — Appendix S1. Supporting Information. [file CLC-44-839-s001.docx]

***APPENDIX***

***ACS Reflective II Investigators***

**British Columbia**

University of British Columbia, Vancouver: Christopher Fordyce, Matthew Bennett, Graham Wong, Sean Virani, Tara Sedlak, Nathan Brunner.

Keary Medical Centre and Foremed Clinic, New Westminster: Razi Khan, Richard Vandegriend, Roger Philipp.

Tri Cities Cardiology, Port Moody: Vineet Bhan.

**Saskatchewan**

Regina General Hospital - Prairie Vascular Research Network (PVRN) - SETFAST Coordinating Centre (SCC), Regina: Payam Dehghani, Andrea Lavoie.

**Ontario**

St Michael’s Hospital, Toronto: Andrew Yan, Akshay Bagai, Kim Connelly, Jeremy Edwards, Howard Leong-Poi.

St Mary’s Regional Cardiac Centre, Kitchener: Hahn Hoe Kim.

Halton Healthcare, Oakville: Michael Heffernan, Sean Jedrzkiewicz, Michelle Paikin, Qin Li.

Sunnybrook Health Sciences Centre: Mina Madan, Harindra Wijeysundera, Dennis Ko, Shyam Radhakrishnan, Shaheeda Ahmed.

St Joseph’s Healthcare, London: Neville Suskin, Robert McKelvie, Andrew Mathew.

University Hospital/London Health Sciences Centre, London: Shahar Lavi.

Scarborough Cardiology Research Associates, Scarborough: Ashok Mukherjee, Saleem Kassam, Joe Ricci, Ram Vijayaraghavan, Nisha D'Mello, Paul Galiwango, Amir Janmohamed.

**Quebec**

McGill Health Centre: Thao Huynh, Montreal.

Institut universitaire de cardiologie et de pneumologie de Quebec - Hôpital Laval, Quebec: Jean-Pierre Déry, Paul Poirier, Guy Proulx, Jean-Michel Paradis, Tomas Cieza, Bernard Cantin.

**New Brunswick**

New Brunswick Heart Centre, Saint John: Sohrab Lutchmedial, Greg Searles, Peter Fong, Robert Teskey, Vernon Paddock, Jaroslav Hubacek, Colin Barry.
